# Nutritional service needs of pregnant and lactating adolescent girls in Trans-Mara East Sub-County, Narok County: focus on access and utilization of nutritional advice and services

**DOI:** 10.1186/s12884-019-2391-7

**Published:** 2019-07-05

**Authors:** David Omondi Okeyo, Sussy Gumo, Elly O. Munde, Charles O. Opiyo, Zablon O. Omungo, Maureen Olyaro, Rachel K. Ndirangu, Nanlop Ogbureke, Sophie Efange, Collins Ouma

**Affiliations:** 1 Kenya Nutritionists and Dieticians Institute, P. O. Box 20436-00100, Nairobi, Kenya; 2 School of Arts and Social Science, Department of Religion, Theology and Philosophy, Maseno University, Maseno, Kenya; 3 Christian Aid-UK, P. O. Box 13864-00800, Nairobi, Kenya; 4 Christian Aid-UK, 35 Lower Marsh, London, SE1 7RL UK; 5 School of Public Health and Community Development, Department of Biomedical Science and Technology, Maseno University, Maseno, Kenya

**Keywords:** Nutritional needs, Adolescent, Lactating, Pregnant, Kenya

## Abstract

**Background:**

An understanding of the association between adolescent nutrition, adolescent pregnancy and their quest for healthcare services may elucidate a basis for intervention and formulation of programs that enhance post-partum and increase the lifespan of the newborn, improve the quality of life and bridge morbidity, mortality and healthcare-associated cost. However, the nutritional needs of pregnant and lactating adolescent girls aged below 10 years resident in Trans Mara East Sub-County, Kenya remained unestablished. The objective of this study was to assess the nutritional needs of pregnant and lactating adolescent girls (under 19) when accessing and utilizing nutritional advice and services in Trans-Mara East Sub-County, Narok County.

**Methods:**

The study adopted a cross-sectional approach that employed mixed methods with both quantitative and qualitative research approaches. Cochran formula was applied to arrive at a minimum of 291 households. Probability proportionate to size sampling techniques using cluster and simple random methods were used to practically access adolescents who are pregnant or lactating. Data was collected using questionnaires, in-depth interview and Focus Group Discussion. Quantitative data was analyzed descriptively using frequencies and inferentially using odds ratio and z-test. Framework analysis was employed to analyze qualitative data. *p* ≤ 0.05 was considered statistically significant.

**Results:**

The study revealed that access of pieces of nutritional-related advice represented by 67.8% was significantly higher than expected frequency of 50%. Nutrition supplementation, food fortification or blending and complementary feeding were significantly below the expectant frequency (*p* < 0.01) of 50%. Nutrition service areas such as provision and collection of vitamin A and IFAS were significantly lower than expected frequency (*p* < 0.01).

**Conclusions:**

The most widely utilized were nutrition services that falls within the preventive-focused services followed by curative-focused services. Nutritionist and nurse more likely to increase overall utilization of nutrition services.

**Electronic supplementary material:**

The online version of this article (10.1186/s12884-019-2391-7) contains supplementary material, which is available to authorized users.

## Background

Adolescence is a stage where puberty sets and a huge window of opportunity opens up in which they require increased nutritional needs. There are hormonal changes due to the onset of puberty, increased protein, energy, iron, and calcium requirements. It is apparent that children normally gain up to 50% of their respective adult weight, skeletal mass and acquire close to 20% of their height during adolescence [1].

Optimal nutrition becomes essential to attain full growth potential. Having a nutritional deficiency at this formative stage of life can be detrimental to the individual’s future health and even the offspring. For instance, failure to partake a nutritious diet at this interval can lead to retarded sexual maturation and slowed physical growth [1, 2]. When adolescence is confronted with pregnancy, the nutrition requirements further becomes more demanding. Pregnancy presents another special stage in life that has the potential to positively impact on maternal health and that of the preceding generation. Adequate nutrition is imperative to meet the added demands of nutrients for the mother’s body, that of the growing fetus and instills a strong biological basis for the present and coming health, productivity and well-being of the mother [3].

Other investigators further demonstrate that devastating effects of poor nutrition status at adolescent into motherhood, maternal body conformation, altered metabolism and supply of nutrients to the placenta can enhance positively or negatively the fetal development, growth and is interrelated to pregnancy outcome. Moreover, the relationship between nutrition status and pregnancy at adolescent is complex and attributed to different biological, economic, demographic and social factors which vary widely depending on the population in the interplay [3].

An understanding of the association between adolescent nutrition, adolescent pregnancy and their quest for healthcare services may elucidate a basis for intervention and formulation of programs that enhance post-partum and increase the lifespan of the newborn, improve the quality of life and bridge morbidity, mortality and healthcare-associated costs [4]. Whereas these windows of opportunities are critical, it turns out to be vital that adolescent girls should be singled-out to be able to halt the rotational cycle of malnutrition. This is extremely critical for the projected 10 million girls below 18 years that get married every year [5] and the 16 million adolescent girls who give birth each year [6]. In Kenya, the available data from Kenya Demographic Health Survey (KDHS) 2014 does not disaggregate data and thus do not necessarily provide specific information on adolescents aged 10–19 years. Yet, these adolescents face many risks and challenges as pertaining to their nutrition, health and education status.

In Narok County, 40% of girls aged 15–19 years have begun child bearing, almost two times higher than the Kenyan national level (18%), yet to date, no study has established the unmet needs of pregnant and lactating adolescent girls (aged 10–19 years) in accessing and utilizing nutritional advice and services in Trans Mara East Sub-County within Narok County, Kenya. In this region of Trans Mara East Sub-County, there is increased malnutrition due to rising population (attributable to high teenage pregnancies and low education levels) with minimal access to the existing nutritional services. The nutrition services and needs of adolescent is not yet fully explored to lay foundation for interventions. It is against this background that the current study focused on elucidating the needs of pregnant and lactating adolescent girls (aged 10–19 years) in accessing and utilizing nutritional advice and services in Trans Mara East Sub-County within Narok County, Kenya.

## Methods

### Study setting and research design

This study was conducted with within Narok County where 40% of girls aged 15–19 years have begun child bearing, a figure almost two times higher than the national level (18%). Specifically, 7.4% are pregnant with their first child and 33% have ever given birth as compared to the national levels of 3.4 and 14.7%, respectively. These statistics are supported by the risks facing adolescents in Kenya which include but not limited to: high HIV infections, particularly among girls (16% of people living with HIV are aged 10–24 years); high teenage pregnancies (18%); early marriages (11%) for older adolescents (15–19 years); persistent female genital mutilation (11%); high rates of anaemia (41%) among pregnant adolescents; high number of adolescents exposed to sexual violence (11%) and physical violence (50%) as well as low secondary school attendance with a net ratio of 47%. All these risks perpetuate further the vulnerability of this age group to a healthy life.

The study was carried out in Trans Mara East Sub-County within Narok County. Trans Mara East Sub-County was purposively selected since it is the smallest in size (275.4 km^2^), among the four sub-counties in Narok County and had the highest prevalence of teenage pregnancies based on previous survey (Christian Aid, 2018 unpublished data). To achieve the objectives of this formative study, a cross-sectional study employing concurrent mixed methods approaches with both quantitative and qualitative research techniques was applied.

### Study population and sampling technique

#### Population of study

The primary study population comprised of all pregnant and lactating adolescent girls (aged 10–19 years old) resident in Trans Mara East Sub-County, assuming that the prevalence of pregnant and lactating mothers was 50% within the entire Trans Mara East Sub-County, from which a sample was drawn. Pregnant adolescent would be eligible for the study when they were three months pregnant and lactating adolescent girls would be eligible if they had children ≤24 months.

#### Sample size determination for quantitative approach

Sample size was determined using the Cochran formula [7], which allowed for calculation of an ideal sample size given a desired level of precision, desired confidence level, and the estimated proportion of the attribute present in the population. The following formula was applied;

$$ n=\frac{(pq){z}^2}{e^2} $$

Where: n = minimum sample size (for population > 10,000) required.

Z = the standard normal deviate at the required confidence level, (set at 1.96 corresponding to 95%, Confidence level adopted for this study).

p = population proportion estimated to pregnant/ lactating is 50%.

q = 1-p.

e = the degree of accuracy required (usually set at 0.05).

$$ n=\frac{\left(0.5\times 0.8283\right){1.96}^2}{0.05^2} $$

*n* = 384 adolescents + 10% non-response.

Since the Z-value was set at 1.645 corresponding to 90% confidence level, the minimum sample size was:

$$ \frac{\left(0.5\times 0.5\right){1.645}^2}{0.05^2} $$

*n* = 271 adolescents + 10% non-response.

=292 adolescents.

The final sample size obtained was 337 adolescents who were either pregnant or lactating. Proportionate distribution was done across 25 clusters equivalent to villages and by adolescent status (i.e. pregnant or lactating).

#### Test for sample size adequacy

Based on the above formula, the minimum sample size at 90% confidence was 292 pregnant and lactating adolescents. However, given the nature of the questionnaire where 90% of key variable measures were based on 5 point-Likert scale, descriptive test for sample size adequacy using Kaiser-Mayor Olkin and Batt-test of sphericity generated by principle axis factoring, was applied to test for sample size adequacy giving way for subsequent statistical tests.

#### Sampling procedure

Cluster sampling was appropriate under the assumption given the existing wards and villages. Probability sampling techniques using cluster and simple random methods was used to practically access adolescents who were either pregnant or lactating. An enumerator covered at least one village in a day to administer at least 8 questionnaires at random. Each enumerator moved to the center of a village selected for the day and began by facing North direction. After that, eight papers representing North, North East, North West, East, South East, South, South West and West, were randomized and one picked to inform the direction to walk. Once direction was picked, an enumerator walked on a straight line to the next household until he/she reached a household with an eligible adolescent. Once the first adolescent was interviewed the enumerator again stood at the door of the just completed house facing North again and picked a direction from the pieces of papers randomized. The enumerator again walked to the next household. This process was repeated throughout the day until all eight adolescents were interviewed. An enumerator that reached the end of the village before completing the numbers required would go back to the center of the village and randomly select a new direction to walk to. In case an enumerator double-selected the previous household, that household was passed until another eligible adolescent was reached. Each time an enumerator strived to interview at least one adolescent who is pregnant or lactating in the ratio of 3:7 interchangeable along the walk.

### Methods of data collection tools and process

Quantitative data was collected using adolescent questionnaire targeting critical indicators of access and utilization (See Additional file 1). The questionnaire included *indicators of access* such as Advice on Healthy diet/diet diversity; exclusive breastfeeding; nutrient supplementation; food fortification and blending; and appropriate complementary feeding. Services targeted in the questionnaire included, Provision and collection of IFAS; Vitamin A supplementation for the child; sexual and reproductive health sensitive to nutrition e.g. family planning; Basic environmental hygiene, and disease prevention e.g. provision of ITNs; Basic personal hygiene; Regular nutrition assessment both at antenatal and post-natal; Child growth monitoring at post-natal care; Nutrition referral for critical malnutrition episodes; Nutrition support e.g. mother-to-mother support; Nutrition supplements e.g. ready to use therapeutic/Supplementary foods RUTS/RUSF; Regular follow-ups on utilization of services e.g. through community strategy programmes; and lactation management and processes e.g. normally done using lactation charts pathways.

The questionnaire was administered to each respondent by an enumerator for a period of about 45 min. Both open-ended questions and closed-ended questions were used. The questionnaire was administered to adolescents aged 10–19 years who were either pregnant or lactating. The questionnaire Interview method was employed to gather data on the needs of pregnant and lactating adolescent girls (aged 10–19 years) in Trans Mara East Sub-County in accessing and utilizing nutritional advice and services and mapping out current needs of pregnant and lactating adolescent girls (aged 10–19 years) in Trans Mara East Sub-County in Narok County and how they are currently met.

Qualitative data was collected using Focus Group Discussions (FGD) guide and in-depth interview method. Three focus groups targeting Community Health Volunteers, Parents and Mother-to-Mother Support Group were conducted to understand issues surrounding nutrition needs of the adolescent girls who are pregnant or lactating. Each target group was made up of 6–10 members to engage in free discussions. In-depth interview was conducted to get in-depth information from adolescents who are pregnant and lactating. This method assisted in gathering data on the needs of pregnant and lactating adolescent girls (aged 10–19 years) in Trans Mara East Sub-County, Narok County in accessing and utilizing nutritional advice and services and to map out current needs of pregnant and lactating adolescent girls (aged 10–19 years) in Trans Mara East Sub-County, Narok County. In addition, information on the intervention measures that need to be considered for the needs to be met within the context of the Sub-County, was also collected. The approach was appropriate for getting more information that may not be shared in groups. In-depth interviews were conducted until saturation. At least 6 cases of lactating and 6 cases of pregnant adolescents were selected by convenience among 25 clusters, and were engaged for in-depth interview. Major questions of FGDs and in-depth interview included who provides nutrition advice and services for adolescent pregnant/lactating for adolescent pregnant/lactating at the health facility. A mention of some of the facilities nutrition pieces of advice/services provided for adolescent pregnant/lactating mothers; How these facilities are accessible and how the nutrition advise information is conveyed to the adolescent pregnant/lactating whenever they visit a facility to seek services, and finally we established the level of satisfaction with the nutrition and health information provided to the adolescents.

### Statistical analyses

*Quantitative data analysis* adopted use of descriptive and inferential statistics. Descriptive statistics was used to characterize different frequencies. Z-test for single proportions was used to test for significant difference between the actual frequencies and expected frequency. Expected frequency was set at 50% for dichotomized data and 100/n percent for data that had more than two options. Principal Axis Factoring was used to establish the access pattern as well as generating *Batt*-scores for further modeling especially for indicators that were fitted into access and utilization models to determine cause and effect. *p* ≤ 0.05 was considered statistically significant.

*Qualitative Data Analysis* on the other hand adopted the use of *Framework analysis* for both in-depth interviews and Focus Group Discussions. One key advantage with this framework analysis is that although it uses a thematic approach, it allows themes to develop both from the research questions and from the narratives of research participants. The process of data analysis began during the data collection, by skillfully facilitating the discussion and generating rich data from the interviews and FGDs, while complementing them with the observational notes and typing the recorded information. This stage was followed by familiarization with the data, which was achieved by listening to voice records, reading the transcripts in their entirety several times and reading the observational notes taken during and after the interview and/or FGDs. The aim was to immerse in the details and get a sense of the interview as a whole before breaking it into parts. The next stage involved identifying a thematic framework, by writing *narrative memos* in the margin of the text in the form of short phrases, ideas or concepts arising from the texts and beginning to develop categories. At this stage, descriptive statements were formed and an analysis carried out on the data under the questioning route. The third stage, *indexing*, comprised sifting the data, highlighting and sorting out quotes and making comparisons both within and between cases. The fourth stage, *charting*, involved lifting the quotes from their original context and re-arranging them under the newly-developed appropriate thematic content.

## Results

### Access and utilization of nutrition advise and services among adolescent who are lactating and pregnant

Prior to establishing access and utilization of nutrition advice and services, access of pieces of nutritional-related advice was assessed among pregnant and lactating adolescents. Results showed that majority received at least one piece of advice with a significant frequency of 67.8% higher than expected frequency of 50%. However, a focus on five specific advice domains revealed that pieces of advice to both pregnant and lactating adolescent were majorly surrounding healthy diet/diet dietary intake (48.4%) and exclusive breastfeeding (41.8%). The two domains were below the 50% expected frequency though at insignificant level (*p* > 0.05). Nutrition supplementation, food fortification or blending and complementary feeding were significantly below the expected frequency of 50% (*p* < 0.01) (Table 1).

**Table 1:** Distribution of adolescent who are lactating and pregnant by frequency of nutrition advice received

Advise Need indicators (*n* = 335)	Frequency	Percent	z-value at 50% expected frequency	*P*-value
Proportion who received nutrition advice in the past 3 months	227*	67.8	4.68	< 0.05
*Advice Domain*				
Healthy diet/diet diversity	162	48.4		
Exclusive breastfeeding	140	41.8		
Nutrition supplementation	68**	20.3	−8.1	< 0.05
Food fortification and blending	43**	12.8	−10.4	< 0.05
Complementary feeding	87**	26.0	−6.4	< 0.05

* *p* < 0.05; ** *p* < 0.01 based on z-test effect size

A further screening of 14 regular services offered to pregnant and lactating adolescent mothers revealed that 77.0% received at least one service, which included nutrition education and counseling (53.4%) and regular nutrition assessment (50%). The child growth monitoring (48.1%) was slightly lower than expected frequency of 50%, however, this was not statistically significant (*p* > 0.05). Other critical nutrition-specific service areas screened for utilization included provision and collection of IFAS (45.7%), vitamin A supplementation (31.6%), efforts on regular follow ups on utilization of services (27.2%), deworming (22.7%), mother-to-mother support (22.4%), nutrition supplementation (11.6%), nutrition referral (5.1%) and lactation management and process (3.3%). These categories of services were significantly lower than expected frequency (*p* < 0.01 in all cases) (Table 2).

**Table 2:** Distribution of adolescents who are lactating and pregnant by frequency of nutrition services received

Service Need indicators (*n* = 335)	Frequency	Percent	z-value at 50% expected frequency	*P*-value
Proportion who received any nutrition service in the past 3 months	258***	77.0	7.26	< 0.05
*Service domain*				
Provision and collection of IFAS	153	45.7		
Nutrition education and counselling	179	53.4		
Deworming	76	22.7		
Vitamin A supplementation for the child	106*	31.6	−4.85	< 0.05
Sexual and reproductive health sensitive to nutrition e.g. family planning	141	42.1		
Basic personal hygiene	223	66.6		
Regular nutrition assessment both at antenatal and postnatal	168	50.1		
Child growth monitoring at postnatal care	161	48.1		
Nutrition referral for critical malnutrition episodes	17**	5.1	−13.0	< 0.05
Basic environmental hygiene and disease prevention e.g. provision of ITNs	251	74.9		
Nutrition support e.g. mother to mother support	75**	22.4	−7.43	< 0.05
Nutrition supplements e.g. ready to use therapeutic/supplementary foods RUTFs/RUSFs	39**	11.6	−10.46	< 0.05
Regular follow-ups on utilization of services e.g. through community strategy programmes	91**	27.2	−6.06	< 0.05
Lactation management and processes e.g. normally done using lactation charts pathways	11**	3.3	−13.67	< 0.05

* *p* < 0.05; ** *p* < 0.01 ***; *p* < 0.001 based on z-test effect size

Discussions with adolescent mothers, CHVs and parents revealed ongoing nutrition advice and counseling within the community and at family level. The CHVs are trained on how adolescents who are lactating and pregnant should be taken care of nutritionally. It appeared in the discussions and in particular, with parents that they only knew about medical doctors and CHVs. This means that any health personnel who attended to the adolescent’s needs were generally referred to as a *doctor* implying that the role of other cardres were improperly perceived. There is thus a possibility that a nutritionist or a nurse could easily be referred to as a ‘doctor’ as captured in the following FGD quote.

*“R10-always when they come to hospital, we always do follow –up as CHVs…….”*

*R3- as CHV we are always trained on how to help those adolescents lactating and pregnant …*

*R3- when we go to the hospital, we meet guiding and counselling doctor who assist us…*

*R1- I think the person who provides is ‘Daktari’, also CHVS always give them direction on where to access.*

### Facility-related factors in relation to access to pieces of advice and utilization of nutrition services

The study further focused on support systems for access to health services by exploring service provider pattern, facility sources, distance to source and methods of conveying information to adolescents (both pregnant and lactating). It appeared that in Trans-Mara East Sub-County, nurses are the main providers of nutrition advice needed (55.2%), followed by the CHVs (24.2%), then nutritionists (21.5%), clinical officers (17.6%), social worker (3.0%) and pharmacist (1.8%) in that order. A test of significance revealed that nutritionists who are supposed to be the key provider registered a significantly lower than expected frequency (*p* < 0.01) of 50% as a service provider frequently used by the adolescents. Nurses on the other hand, registered higher but non-significant proportion than expected frequency of 50% (*p* > 0.05).

Within facility-based sources of information, nutrition advises and services were significantly sought at dispensary level (67.2%) higher than expected frequency of 50% (*p* < 0.01), followed by school (22.1%). Further z-test indicated that other than dispensaries, other facilities recorded significantly lower than expected frequency of 50% as sources of nutrition information.

On matters of distance to facility, majority accessed them within 1-5 km distance (67.2%) which is significantly higher than 25% expected frequency (*p* < 0.01). Generally, the adolescents who are pregnant or lactating dominantly receive information on nutrition and health through face-to-face interaction with service provider (87.2%). Radio broadcasting or TV also become useful methods through which information is relayed at 32.8% (Table 3).

**Table 3:** Distribution of adolescent who are lactating and pregnant by providers, sources distance and methods of conveying information on nutrition advice or services received

	Frequency	Percent	z-value at 50% expected frequency	*P*-value
Nutrition advise and service providers (*n* = 335)				
Nutritionists	72**	21.5	−7.7	< 0.05
Nurse	185	55.2		
Clinical officer	59**	17.6	−8.86	< 0.05
CHVs	81**	24.2	−6.9	< 0.05
Community Development Social Worker	10**	3.0	−13.78	< 0.05
Pharmacists	6**	1.8	−12.68	< 0.05
Facility type as Source of Nutrition advise and service providers (*n* = 335)				
Public dispensaries	225	67.2		
Private clinic	33	9.9		
Private hospital	6	1.8		
Public hospital	3	.9		
Public health centre	33	9.9		
CBO and NGO health	19	5.75		
FBO project	17	5.1		
At school	74	22.1		
Distance to Source of Nutrition advise and service providers (*n* = 335)				
< 1 km	63	18.8		
1–5 km	225*	67.2	4.52	< 0.05
5-10 km	40	11.9		
> 10 km	7	2.1		
Methods of conveying Nutrition advise by service providers (*n* = 335)				
IEC materials e.g. brochures, leaflets etc. IEC materials e.g. brochures, leaflets etc.	18	5.4		
Bulk SMS	0	0.0		
Internet links	0	0		
Face to face	292*	87.2	10.4	< 0.05
Video clips	2	0.6		
Social media e.g. WhatsApp and Facebook pages	11	3.3		
Through radio broadcasting or TV	110	32.8		

* *p* < 0.05; ** *p* < 0.01 based on z-test effect size; *CBO* Community-Based Organizations, *NGO* Non-Governmental Organization, *FBO* Faith-Based Organizations, *IEC* Information, Education and Communication materials, *SMS* short messaging systems

The discussants, especially the adolescent mothers, re-affirmed their frequent visit to clinics for their children to undergo growth monitoring. The CHVs also do follow-ups especially during immunizations to attend to adolescents who are mothers. Again, information relay was confirmed by CHVs as being through follow-ups at household level on one-on-one basis and through community forums mainly described as ‘*matangazo’* (frequent community forum on matters of health). This is further captured in the following FGD:

*R7-we as volunteers we usually go for follow –up to ensure they come to last schedule for immunizations….*

*R6-I go to visit clinic and they see the presentation of the child…*

*R7- At hospital the doctor assists also in weighing children and attending to their complications like diseases.*

*R4-the way they get information is through us CHVs because we always do in –depth follow–up in community and also the community forum is always given matangazo there.*

### Nutrition services utilization pattern

The study further assessed 7 [Iron and Folic Acid Supplements (IFAS), regular nutrition assessment, practice of diet quality, use of Insecticide-Treated Nets (ITNs) and regular visit for nutrition education and counseling, Ready-to-Use Therapeutic Supplements/Ready to Use Supplementary Feeds (RUTS/RUSF and vitamin A supplementation)] service areas of nutrition services based on adherence rating mechanisms to explore adherence pattern (Table 4). Based on principal axis factor loadings, the 7 adherence items generated two-factor loading categories both accounting for a total of 42.2% within the service domain.

**Table 4:** Utilization of critical nutrition services based on factor loading

Adherence rating	Service Domain	
	Factor 1	Factor 2
Collection and use of IFAS	0.603	
Regular Nutrition Assessment	0.824	
Practice of quality diet	0.533	
Use of RUTS/RUSF		0.431
Vitamin A supplementation for the child (Applicable For Lactating Mothers)		0.733
Use of ITNs	0.439	
Regular visit for nutrition education and counseling	0.679	
Variance based on Rotated sum of Squared Loading	29.897	12.346

Minimum Commonality at Eigen value of 1 = 0.4

Factor 1 which accounted for 29.9% of service domain loaded five items in the rotated matrix based on Eigen value = 1. These included collection of IFAS (cumulative, cum = 0.60), regular nutrition assessment (cum = 0.82), practice of diet quality (cum = 0.53), use of ITNs and regular visit for nutrition education (cum = 0.44) and counseling (cum = 0.68). This category of service adherence was labelled *“preventive focused services”* with nutrition assessment being the most utilized followed by regular visit for nutrition education and counseling, based on community coefficient.

Factor 2 which accounts for 12.3% of nutrition service adherence domain loaded 2 items in the rotated matrix. The two items included use of RUTS/RUSF (cum = 0.40) and vitamin A supplementation (cum = 0.73). This category of service adherence was labelled *“curative focused services”*.

## Discussion

Access and utilization of nutrition services among adolescents is a complex phenomenon that is affected by a number of factors. Studies carried out in developing countries underscore the importance of socio-economic factors and the environment of service delivery as key determinants of utilization of nutrition services among the adolescents [4, 8, 9]. Other factors such as distance from the health facility, level of education, lack of autonomy and the power of decision-making, cultural norms, religion and the quality of service delivered have also been associated with the level of access and utilization of nutrition services among the adolescents [10–12]. The current study assessed the nutritional needs of pregnant and lactating adolescent girls (under 19) when accessing and utilizing nutritional advice and services in Trans-Mara East Sub-County, Narok County, Kenya.

### Access to nutrition advice and services in trans Mara east Sub-County, Narok County

The study very significantly demonstrated low coverage of 337 adolescents for supplementation, exclusive breastfeeding, food fortification and blending as well as complementing feeding. This implies that delivery of nutrition advice to pregnant and lactating adolescent is weak in Trans Mara East Sub-County, Kenya. This scenario as previously noted, may lead to poor nutrition status for the adolescents and could interfere with productivity and well-being of the mother [3]. Inadequate access to good information on healthy diet for adolescents who are pregnant may also lead to preterm deliveries, low weight babies and anemia [13].

Low coverage of complementary feeding-focused-advice which was clearly evident in this study would pose a challenge to children during early stages of life especially within their first 1000 days [14]. This is because growth retardation may be experienced immediately after 6 months of exclusive breastfeeding and may continue for some time [15]. Pieces of advice on effective complementary feeding should be provided to all adolescents who are lactating to prevent any possible shock during initiation of complementary feeding or weaning. Failure to mitigate this aspect would create an immense risk to adolescents’ proper utilization of services.

Supplementation, food fortification and blending also play a key role during pregnancy and lactation. This study revealed low coverage on pieces of advice given to promote supplements, fortification and blended food products. This could be a high risk to malnutrition for the mother and child. Fortification of commonly used foods serve as vehicles and presents opportunities for increasing nutrient intake for infants [16]. Lack of inadequate knowledge on this intervention mechanism may be a high risk to an adolescent who is pregnant or lactating, thus potentially perpetuating poor nutrition of children born to them. Increasing knowledge of a mother in supplementation programmes may therefore also contribute to child growth by proxy. Low coverage through advice lead to poor knowledge of the mother and hence heighten the children’s risk to poor growth. Utilization of services that support optimal nutrition for the adolescent also displayed low coverage within Trans Mara East Sub-County. The most affected areas of services were Vitamin A supplementation, nutrition referrals, nutrition support, nutrition supplements, community follow ups and lactation management and processes. The performance of these indicators of access to services are critical for an adolescent nutrition situation. High coverage is needed to be certain that the adolescent received adequate nutrition-specific care to enjoy quality of life [4].

### Utilization of nutrition services in trans Mara east Sub-County, Narok County

In the current study, utilization of services was based on common routine at facility level. Two categories of utilization demonstrated that prevention component had a higher weight than curative component of regular nutrition care process. This is true based on the fact that other treatments are in most cases anchored on assessment outcome. Nutrition assessments are often done to assess the body composition measures or adequacy of diet. Nutrition status of pregnant women is useful in determining pregnancy outcome and through this, a mother would be attended to on the basis of informed evidence with appropriate baseline information on nutritional needs.

Other preventive components such as collection of IFAS, practice of quality diet, nutrition education of counseling and disease prevention only follow with clear assessment outcome. Provision of IFAS is a preventive component of nutrition interventions that has a chance of reducing incident of anemia in mother and low birth weight in neonates [17]. Other service domains such as practice of regular nutrition education, practice of quality diet and use of ITNs are all enhanced in the outcome of assessment. A malnourished adolescent or child born to adolescent would necessitate serious intervention, which in many cases, is tailored in nutrition education, counseling and practice of quality diet. Every so often, disease prevention mechanisms are incorporated mainly to prevent infection as malnutrition lowers immunity, consequently augmenting their risk to infections.

Vitamin A supplementation for children and provision of RUSF are target intervention for high risk cases. These are therapeutic products that only target curative measures. Ready-to-use-Therapeutic-Foods (RUTFs) such as plumpy nuts and therapeutic milks are food alternatives for medical complications such as loss of appetite, severe dehydration, edema, high fever and anorexia. These are common problems in rural regions where RUTFs act as immediate remedy. Availability, access and knowledge on how to use these products can greatly reduce severity of malnutrition [18]. Vitamin A supplementation is significant for eye health, immune function and fetal growth and development. Vitamin A deficiencies result in visual loss exhibited through night blindness and, in children, may intensify the danger of illness and death from childhood infections, including measles and those diarrhea-causing pathogens. Pregnant women become susceptible to vitamin A deficiency especially during the third trimester. At this stage, it is paramount that pregnant girls and women are advised to consume an optimally nutritious diet to reduce chances of deficiency [19].

## Conclusions

This study established that adolescent needs were being met with 67.8% reporting that they received nutrition advice in at least one area of service. However, coverages of critical service domains were still below average. This included advice on healthy diet/diet diversity, exclusive breast feeding, nutrition supplementation, food fortification and blending as well as complementary feeding. The last three domains were significantly below the average and would require much attention. On matters of utilization, the study isolated two domains of utilization of nutrition services. The most widely utilized were nutrition services that falls within the preventive-focused services such as collection of IFAS, regular nutrition visits for counseling and education, practice of quality diet and use of ITNs. The second level of utilization was curative-focused services which were characterized by vitamin A supplementation and use of RUTF/RUSF. Based on qualitative data analyses, nutritionist and nurse were more likely to increase overall utilization of nutrition services.

## Recommendations

1. Since education support given to adolescents somehow showed it as a barrier to low utilization of nutrition services, nutrition programmes targeting pregnant and lactating adolescent should be set up in schools to provide relevant nutrition education, healthy meals and environments.
2. There is need to increase access to adolescents within the community set up through considerable scope for making better use of multiple avenues to reach adolescents, including school-based, health system-based and community-based approaches; marriage registries (where available) could be used to target newly-wed adolescents to improve coverage reaching adolescents with information at community.

## Additional file

Additional file 1: These are the dataset supporting the conclusions of this article which is provided as Additional file 1. (XLSX 166 kb)

## Data Availability

The dataset supporting the conclusions of this article is included within the article as Additional file 1.

## Abbreviations

CHV: Community Health Volunteers
FGDs: Focus Group Discussions
HIV: Human Immunodeficiency Virus
IFAS: Iron and Folic Acid Supplementation
ITNs: Insecticide-Treated Nets
KDHS: Kenya Demographic and Health Survey
KNDI: Kenya Nutritionists and Dieticians Institute
RUSF: Ready-to-use-Supplementary-Foods
RUTFs: Ready-to-use-Therapeutic-Foods
